# SPOP negatively regulates Toll-like receptor-induced inflammation by disrupting MyD88 self-association

**DOI:** 10.1038/s41423-020-0411-1

**Published:** 2020-03-31

**Authors:** Yun-Hong Hu, Yang Wang, Fei Wang, Yan-Ming Dong, Wan-Ling Jiang, Ya-Ping Wang, Xing Zhong, Li-Xin Ma

**Affiliations:** 1 State Key Laboratory of Biocatalysis and Enzyme Engineering, School of Life Sciences, Hubei University, Wuhan, 430062 China; 2 School of Biological and Chemical Engineering, College of Zhixing, Hubei University, Wuhan, 430011 China

**Keywords:** SPOP, MyD88, TLR, inflammation, Toll-like receptors, NF-kappaB

## Abstract

Toll-like receptor (TLR) signaling pathways need to be tightly controlled to avoid excessive inflammation and unwanted damage to the host. Myeloid differentiation primary response gene 88 (MyD88) is a critical adaptor of TLR signaling. Here, we identified the speckle-type POZ protein (SPOP) as a MyD88-associated protein. SPOP was recruited to MyD88 following TLR4 activation. TLR4 activation also caused the translocation of SPOP from the nucleus to the cytoplasm. SPOP depletion promoted the aggregation of MyD88 and recruitment of the downstream signaling kinases IRAK4, IRAK1 and IRAK2. Consistently, overexpression of SPOP inhibited the TLR4-mediated activation of NF-κB and production of inflammatory cytokines, whereas SPOP depletion had the opposite effects. Furthermore, knockdown of SPOP increased MyD88 aggregation and inflammatory cytokine production upon TLR2, TLR7 and TLR9 activation. Our findings reveal a mechanism by which MyD88 is regulated and highlight a role for SPOP in limiting inflammatory responses.

## Introduction

Toll-like receptors (TLRs) are germline-encoded pattern recognition receptors (PRRs) that play central roles in pathogen recognition and elimination.^1^ TLRs have a conserved cytoplasmic signaling Toll/IL-1R (TIR) domain. Binding of pathogen-associated molecular patterns to a TLR triggers innate immune responses through the myeloid differentiation primary response gene 88 (MyD88)-dependent pathway and/or TIR-domain-containing adapter-inducing interferon-β(TRIF)-dependent pathway and initiates downstream signaling events that lead to the production of proinflammatory cytokines and type I interferons (IFNs).^2–4^

All TLRs except TLR3 transduce signals downstream through MyD88. MyD88 is a 296 amino acid (aa) protein that consists of the N-terminal death domain (DD) and the C-terminal TIR domain; the TIR domain is critical for coupling with the TIR domain in receptors, and the DD domain is crucial for the recruitment of downstream kinases.^2,5^ After receptor ligation, MyD88 is recruited to TLRs, which induces the aggregation (dimerization/oligomerization) and activation of MyD88.^6,7^ MyD88 then acts as a platform to assemble myddosomes by recruiting intracellular IL-1R-associated kinase 4 (IRAK4), IRAK1, and IRAK2. Formation of a myddosome leads to the activation of tumor necrosis factor receptor-associated factor 6 (TRAF6).^6,8–10^ TRAF6 further mediates the activation of NF-κB and induction of inflammatory genes.^11^ TLR3 transduces signals through TRIF.^12^ TLR4 is the only receptor that signals through MyD88 to activate NF-κB and TRIF, which activate both NF-κB and IRF3.^13–15^ Although TLRs are essential for the elicitation of protective immune responses against infection, inappropriate TLR responses contribute to chronic inflammation and/or autoimmune diseases. Thus, precise control of TLR signaling is critical for maintaining immune homeostasis.

Because MyD88 is crucial in meditating pathogen-triggered innate immune responses, its activation must be tightly controlled to maintain immune homeostasis. Indeed, several regulators have been demonstrated to modulate MyD88 activation. For example, the E3 ubiquitin ligases Smurf and Nrdp1 have been found to turn off MyD88 activation by targeting MyD88 for ubiquitination and degradation.^16,17^ The deubiquitinase CYLD has been demonstrated to negatively regulate inflammation by suppressing the K63-linked ubiquitination of MyD88.^18^ MyD88s (MyD88 short) has been shown to associate with MyD88 and impair the MyD88-IRAK4 interaction to inhibit TLR signaling.^19,20^ Although much progress has been made in the regulation of MyD88 activation, the molecular mechanism underlying the self-association of MyD88 remains largely unknown.

Speckle-type POZ (pox virus and zinc finger protein) protein (SPOP) is a member of the BTB/POZ protein family, which contains a conserved BTB/POZ protein–protein interaction domain. BTB/POZ proteins can recruit ubiquitin ligases, histone deacetylases, and corepressors for the assembly of complexes that typically modify chromatin conformation and regulate gene expression.^21,22^ SPOP was first identified as a novel nuclear antigen by antibodies from patients with autoimmune disorders.^23,24^ Subsequently, SPOP has been shown to act as an E3 ubiquitin ligase adapter protein and is involved in diverse biological processes, including cell proliferation, differentiation, and apoptosis.^25–27^ Recently, it was demonstrated that SPOP plays a tumor suppressive role in prostate and endometrial cancers and a tumor-promoting role in kidney cancer.^28–31^

In this study, we report that SPOP participates in TLR-mediated signaling. We identified SPOP as a MyD88-associated protein with yeast two-hybrid technology.^32^ SPOP was associated with MyD88 upon TLR4 activation. TLR4 activation also caused the translocation of SPOP from the nucleus to the cytoplasm. Overexpression of SPOP disrupted the self-association of MyD88. Depletion of SPOP increased the aggregation of MyD88 and recruitment of the downstream IRAK kinases and enhanced the expression of inflammatory cytokines upon TLR activation. Our findings suggest that SPOP acts as a negative regulator of TLR-triggered inflammation by disrupting MyD88 self-association.

## Materials and methods

### Reagents and antibodies

Lipopolysaccharide (LPS) (Sigma, St. Louis, MO, USA), peptidoglycan (PGN) (Invivogen, San Diego, CA, USA), Poly(I:C) (Invitrogen, Carlsbad, CA, USA), R837 (Invivogen), CpG (Invivogen), murine IL-1β (PeproTech, Rocky Hill, NJ, USA), SYBR (Bio-Rad, Hercules, CA, USA), TRIzol reagent (TaKaRa, Dalian, China); mouse monoclonal antibodies against Flag (Sigma), HA (Covance, Princeton, NJ, United States), Myc (Proteintech, Rosemont, IL, USA), β-actin (Proteintech), IκBα (CST, Danfoss, MA, USA) and phospho-IκB (Ser32/36) (CST); rabbit monoclonal antibody against MyD88 (CST), phospho-IRAK4 (Ser345/346) (CST) and phospho-IRF3 (Ser396) (CST); rabbit polyclonal antibody against IRF3 (Proteintech), IRAK1 (CST), IRAK2 (CST), IRAK4 (CST), ubiquitin (CST), K48-polyubiquitin (CST), H3 (ABclonal, Wuhan, China), and SPOP (Proteintech) were purchased from the indicated manufactures.

### Constructs

Mammalian expression plasmids for Flag- or HA-tagged TLR4, Mal, SPOP and its mutants, MyD88 and its mutants, and Myc-tagged SPOP, were constructed by standard molecular biology techniques. The Flag-PRA plasmid was kindly provided by Dr. Chen-Ji Wang (Fudan University).

### RNAi experiments

Nonspecific control siRNA and siRNAs for human/mouse SPOP were purchased from GenePharma (Suzhou, China). The following target sequences for the human SPOP cDNA were used: #1:5′-CAACTATCATGCTTCGGAT-3′; #2: 5′-GGTAAAGGTTCCTGAGTGC-3′. The following target sequences for the mouse SPOP cDNA were used: #1: 5′-CCAAGGGAGAAGAAACCAA-3′; #2:5′-GCATACCGTTCTCTGGCTT-3′. The sequence of the nonspecific control was 5′-TTCTCCGAACGTGTCACGT-3′.

### Quantitative PCR (qPCR)

Total RNA was isolated from cells using TRIzol reagent (TaKaRa), and RNA was reverse-transcribed with a fast cDNA synthesis kit (Tiangen). Quantitative real-time PCR was performed with SYBR Green Supermix (Bio-Rad) according to the manufacturer’s instructions. GAPDH was used as a calibrator for normalization. The following gene-specific primer sequences were used: *SPOP*, 5′-GGAAGGCTCC AAACCTCG ACAA-3′ (forward), 5′-AGCGTTCTCCACGGACAGGTTA-3′ (reverse); *TNFA*, 5′-GCCGCATCGCCGTCTCCTAC-3′ (forward), 5′-CCTCA GCCCCCTCTGGGGTC-3′ (reverse); *IFNB1*, 5′-TTGTTGAGAACCTCCTGGCT-3′ (forward), 5′-TGACTATGGTCCAGGCACAG-3′ (reverse); *IL1B*, 5′-CCACA GACCTTCCAGGAGAATG-3′ (forward), 5′-GTGCAGTTCAGTGATCGTACAGG-3′ (reverse); and *IL6*, 5′-TTCTCCACAAGCGCCTTCGGTC-3′ (forward), 5′-TCTG TGTGGGGCGGCTACATCT-3′ (reverse).

### Cell culture

THP-1 cells were maintained in RPMI 1640 (Thermo Fisher, Waltham, MA, USA) containing 10% fetal bovine serum (FBS) (Thermo Fisher), 100 units of penicillin and 0.1 mg/mL streptomycin. RAW264.7 and HEK293 cells were maintained in Dulbecco’s modified Eagle’s medium (DMEM) (Thermo Fisher) supplemented with 10% FBS, 100 units of penicillin, and 0.1 mg/mL streptomycin. Cells were cultured in a 5% (vol/vol) CO_2_ incubator at 37 °C.

### Cell lines and lentiviral gene transfer

Transduction of the pLVX-Flag-SPOP or pLVX-SPOP-M plasmid into wild-type and SPOP-knockout THP-1 cells was performed by lentiviral-mediated gene transfer. Briefly, HEK293 cells plated on 100-mm dishes were transfected with the indicated lentiviral plasmids (8 μg) together with pSPAX2 (8 μg) and pMD2G (4 μg) by calcium phosphate precipitation that lasted for 12 h, and then, the cells were incubated with medium without antibiotics for another 24 h. The recombinant virus-containing medium was filtered with 0.22-μm filter (Millipore) and then added to cultured THP-1 cells in the presence of polybrene (4 μg/mL). The infected cells were selected with puromycin (0.5 μg/mL) while in culture for at least 7 days before additional experiments were performed.

### CRISPR/Cas9-mediated genomic editing

The experiments were performed as previously described.^33^ Briefly, potential guide RNAs (gRNAs) targeting the SPOP gene were analyzed using the CRISPR design tool. Double-stranded oligos were cloned into a lentiCRISPRv2 vector and cotransfected with packaging plasmids (provided by Dr. Yan-Ming Dong, Hubei University) into HEK293 cells in culture for 12 h, and then the cells were incubated with medium without antibiotics for another 24 h. The recombinant virus-containing medium was filtered and then added to cultured THP-1 cells in the presence of polybrene (4 μg/mL). The infected cells were selected with puromycin (0.5 μg/mL) while in culture for at least 10 days before additional experiments were performed. The SPOP gRNA target sequence was 5′-TAACTTTAGCTTTTGCCGGG-3′.

### Subcellular fractionation

THP-1 cells or BMDMs stimulated with LPS or IL-1β for the indicated times were washed with PBS twice. The subcellular fractions were separated by a Nucl-Cyto preparation kit (Applygen, Beijing, China). In brief, the cells were lysed by whipping with a 1-mL syringe forty times in 1 mL of CEB. The homogenates were centrifuged twice at 1000*g* for 5 min to precipitate the cytoplasm. The pellets were washed with NEB and centrifuged twice at 1000*g* for 5 min to precipitate the nuclei.

### Coimmunoprecipitation and immunoblot analysis

For the transient transfection and coimmunoprecipitation experiments, HEK293 cells were transfected for 20 h. The transfected cells were lysed in l mL of NP-40 lysis buffer (20 mM Tris-HCl, pH 7.4; 150 mM NaCl; 1 mM EDTA; and 1% NP-40) supplemented with protease inhibitor cocktail (Roche). For each immunoprecipitation experiment, a 0.4-mL aliquot of lysate was incubated with 0.5 μg of antibody or control IgG and 25 μl of a protein G sepharose slurry (GE Healthcare, Marlborough, MA, USA) at 4 °C for 3 h. The sepharose beads were washed three times with 1 mL of lysis buffer containing 0.5 M NaCl. The samples were fractionated by sodium dodecyl sulfate polyacrylamide gel electrophoresis (SDS-PAGE) and transferred onto polyvinylidene fluoride membranes (Millipore), and subsequent immunoblot analysis was performed with the indicated antibodies. For endogenous coimmunoprecipitation experiments, THP-1 cells or RAW264.7 cells were stimulated for the indicated times. The cells were then lysed and subjected to coimmunoprecipitation and immunoblot analysis as described above.

### Native polyacrylamide gel electrophoresis (native-PAGE)

For immunoblot analysis under native (nondenaturing) conditions, RAW264.7 cells were collected and washed twice with PBS. Then, the cells were lysed in 0.5 mL of native lysis buffer (50 mM Tris-HCl, pH 8.0; 150 mM NaCl; 1 mM EDTA; 1% NP-40; and 0.5% C_24_H_39_O_4_Na) supplemented with protease inhibitor cocktail (Roche) for 1 h on ice. The lysates were centrifuged for 30 min at 15,000 rpm. For native samples, 0.2-mL aliquots of lysate was added to 5× native loading buffer and then subjected to native-PAGE and immunoblot analysis. For denaturing the samples, 0.2-mL aliquots of lysate was added to 5× SDS loading buffer and then boiled for 10 min before being subjected to SDS-PAGE for immunoblot analysis.

### Immunoprecipitation under denaturation conditions and a ubiquitination assay

The cells were lysed in lysis buffer containing 1% SDS and denatured by heating for 5 min. The supernatants were diluted with NP40 lysis buffer supplemented with protease inhibitor cocktail (Roche) until the concentration of the SDS was decreased to 0.1%. The diluted supernatants were subjected to immunoprecipitation with the indicated antibodies, and then the immunoprecipitates and whole-cell lysates were analyzed by immunoblotting with the indicated antibodies.

### Preparations of BMDMs

Bone marrow cells were isolated from the tibias and femurs of wild-type C57BL/6 mice (6–8 weeks of age). For preparation of the BMDMs, the bone marrow cells were cultured for 4 days in 1640 medium supplemented with 10% FBS, 1% penicillin/streptomycin, and 10% conditional medium from M-CSF-L929 cells (provided by Dr. Yan-Ming Dong, Hubei University).

### Glutathione S-transferase (GST) pull-down assay

GST-MyD88 was purified by glutathione agarose beads (GE Healthcare). His-SPOP was purified by Ni-nitrilotriacetic acid agarose (GE Healthcare). These purified proteins were incubated with glutathione agarose beads for 3 h. The beads were washed 3 times with wash buffer (137 mM NaCl, 2.7 mM KCl, 10 mM Na_2_HPO_4_, 2 mM KH_2_PO_4_ and 0.5% Triton X-100), mixed with an equal volume of 2× SDS loading buffer and boiled for 10 min. The inputs/elutions were resolved by SDS-PAGE and analyzed by Coomassie staining and/or immunoblot analysis.

### ELISA

The supernatants of the cell culture medium were analyzed using the indicated enzyme-linked immunosorbent assay (ELISA) kit (Thermo Fisher) following the protocols recommended by the manufacturer.

### Gel filtration chromatography

THP-1 cells were treated with LPS or left untreated for 1 h. The cells were lysed in 1.5 mL of lysis buffer. The lysate was centrifuged for 1 h at 15,000 rpm. The supernatant was recovered and loaded on a Superdex 200 gel filtration chromatography column pre-equilibrated with lysis buffer. The samples were eluted from the column in lysis buffer at a flow rate of 0.5 mL/min and collected in fractions of 0.5 mL. The fractions were precipitated with 20% trichloroacetic acid and analyzed by immunoblotting with the indicated antibodies.

### Statistical analysis

Student’s *t* tests were performed for all experiments. The level of significance is shown in each figure (^∗^*p* < 0.05; ^∗∗^*p* < 0.01).

## Results

### Identification of SPOP as a MyD88-associated protein

MyD88 is a cytoplasmic adapter protein that is critical for TLR signaling.^14^ Previously, we established a human liver protein interaction network based on yeast two-hybrid technology and identified 3484 potential protein interactions, including the SPOP–MyD88 interaction, suggesting that SPOP may be a MyD88-associated protein.^32^ SPOP is an adapter of the E3 ligase Cullin3, containing a C-terminal BTB domain and an N-terminal MATH domain. The MATH domain is involved in the specific recognition of substrates. SPOP plays important roles in the regulation of development and tumorigenesis by mediating the ubiquitination and degradation of multiple substrates.^25,30,31^

**Fig. 1:**
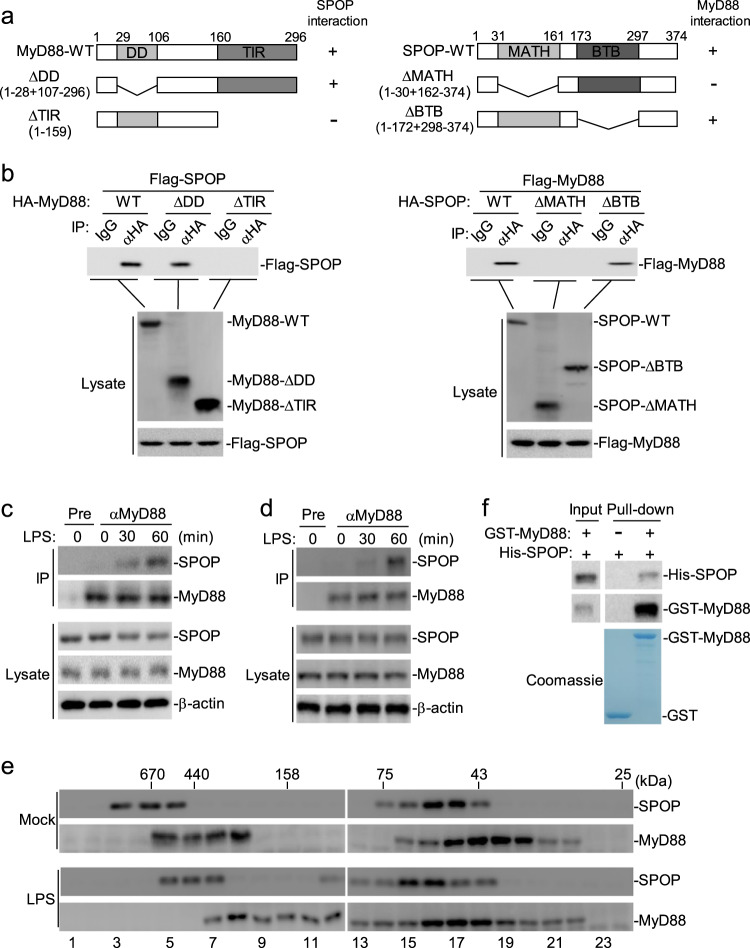
SPOP interacts with MyD88. (**a**) A schematic presentation of full-length MyD88, SPOP and their mutants. (**b**) Domain mapping of MyD88 and SPOP interaction. HEK293 cells (2 × 10^6^) were transfected with the indicated expression plasmids (5 μg each). Coimmunoprecipitation were performed with anti-HA or control mouse lgG. (Upper) The immunoprecipitates were analyzed by immunoblot with anti-Flag. (Lower) Expression of the transfected proteins were analyzed by immunoblots with the indicated antibodies. (**c**) Endogenous association of MyD88 with SPOP in THP-1 cells. The THP-1 cells (1 × 10^8^) were left untreated or treated with LPS (100 ng/mL) for the indicated times. Coimmunoprecipitation and immnoblot analysis were performed with the indicated antibodies. (**d**) Endogenous association of MyD88 with SPOP in RAW264.7 cells. The RAW264.7 cells (1 × 10^8^) were left untreated or treated with LPS (100 ng/mL) for the indicated times. Coimmunoprecipitation and immnoblot analysis were performed with the indicated antibodies. (**e**) Gel-filtration analysis of complexes containing SPOP and MyD88. The THP-1 cells (1 × 10^8^) were treated with LPS for 1 hour or left untreated before lysis. Cell lysates were analyzed by size-exclusion chromatography on Superdex 200 column. The individual fractions were analyzed by Western blots with anti-SPOP and anti-MyD88, respectively. (**f**) SPOP interacts with MyD88 in vitro. Purified GST or GST-MyD88 was used to pull down purified His-SPOP. Proteins bound to the beads were analyzed by immnoblot analysis with the indicated antibodies (Upper). The purified protein were staining with Coomassie brilliant blue (Lower).

To confirm the association between SPOP and MyD88, we performed transient transfection and coimmunoprecipitation experiments with the HEK293 cells. The results indicated that SPOP interacted with MyD88 in the mammalian overexpression system (Fig. 1a, b). Domain mapping experiments indicated that the MATH domain of SPOP and the C-terminal TIR domains of MyD88 were important for their interaction (Fig. 1a, b).

TLR4 is the first-identified and best-studied member of the TLR family.^3^ Since MyD88 is critically involved in TLR4 signaling, we next determined whether endogenous SPOP could associate with MyD88 in TLR4 signaling. As shown in Fig. 1c, endogenous coimmunoprecipitation experiments indicated that SPOP was not associated with MyD88 under physiological conditions. However, their association was readily detected upon LPS (a ligand for TLR4) stimulation in human monocyte THP-1 cells. LPS stimulation also induced an endogenous association between SPOP and MyD88 in mouse macrophage RAW264.7 cells (Fig. 1d). In our gel filtration experiments, we found that SPOP was eluted in complexes of more than 440 kDa and in fractions between 43 and 75 kDa with a peak at approximately 50 kDa, whereas MyD88 mainly coeluted with the low-molecular weight SPOP complexes from the untreated cells (Fig. 1e). Interestingly, both SPOP and MyD88 shifted to show overlapping moderate molecular weight fractions, between 75 and 158 kDa, upon LPS stimulation (Fig. 1e). Finally, we investigated the interaction between SPOP and MyD88 by in vitro precipitation experiments using purified recombinant proteins. His-tagged SPOP was readily precipitated by GST-tagged MyD88 but not by GST, which was used as a control (Fig. 1f), which indicated a direct interaction between SPOP and MyD88. Together, these results indicate that LPS stimulation triggers a direct association between SPOP and MyD88.

### TLR4 activation causes the translocation of SPOP to the cytoplasm

**Fig. 2:**
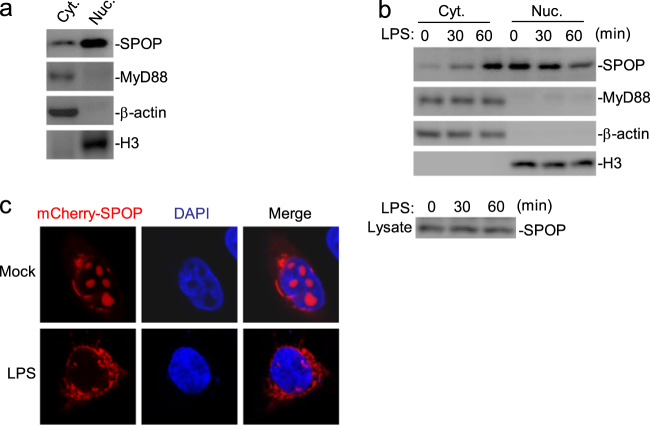
SPOP is translocated to the cytoplasm upon LPS stimulation. (**a**) SPOP was expressed both in the cytoplasm and the nucleus. Cytoplasmic and nuclear fractions from THP-1 cells (3 × 10^7^) were isolated, equilibrated to equal volumes and analyzed by immunoblots with the indicated antibodies. (**b**) Cell fractionation and immunoblot analysis of the subcellular fractions. BMDMs (3 × 10^7^) were treated with LPS (100 ng/mL) for the indicated times. Cell fractionations were performed; the fractions were equilibrated to equal volumes and analyzed by immunoblots with the indicated antibodies (Upper). The whole cellular levels of SPOP upon LPS stimulation were analyzed by immunoblot with anti-SPOP (Lower). (**c**) Confocal microscopy of the cellular localization of SPOP. HEK293 cells (1 × 10^5^) were transfected with expression plasmids for TLR4-Flag (100 ng), MD2-Flag (50 ng) and mCherry-SPOP (50 ng). Twenty hours after transfection, cells were treated with LPS (100 ng/mL) or left untreated for 1 hour, and then fixed with 4% paraformaldehyde and subjected for confocal microscopy. Cyt, cytoplasm; Nuc, nucleus.

Recently, it was demonstrated that SPOP is located in both the cytoplasm and nucleus, although it was previously considered a nuclear protein.^34^ Because SPOP is associated with MyD88 following TLR4 activation, we sought to determine whether SPOP is translocated to the cytoplasm after TLR4 activation. We isolated nuclear and cytoplasmic fractions and found that SPOP was mainly located in the nucleus, and only a small amount of SPOP was detected in the cytoplasm (Fig. 2a). Interestingly, in the THP-1 cells, upon TLR4 activation, the cytoplasmic level of SPOP was increased, whereas the nuclear SPOP level was decreased, with the total level of SPOP unchanged (Supplementary Fig. S1a), which suggested that TLR4 activation caused the translocation of SPOP to the cytoplasm. As previously reported, MyD88 was detected only in the cytoplasm with or without stimulation (Supplementary Fig. S1a).^14^ We also obtained consistent results in mouse bone marrow-derived macrophages (BMDMs) (Fig. 2b). Moreover, we found that IL-1β stimulation caused the translocation of SPOP to the cytoplasm in the BMDMs (Supplementary Fig. S1b). Confocal microscopy experiments further indicated that TLR4 activation caused marked translocation of SPOP to the cytoplasm (Fig. 2c), which was likely originated from the nucleus because TLR4 activation caused a decrease in SPOP in the nucleus. These results suggest that TLR4 activation causes translocation of SPOP to the cytoplasm.

### Overexpression of SPOP inhibits TLR4-mediated signaling

**Fig. 3:**
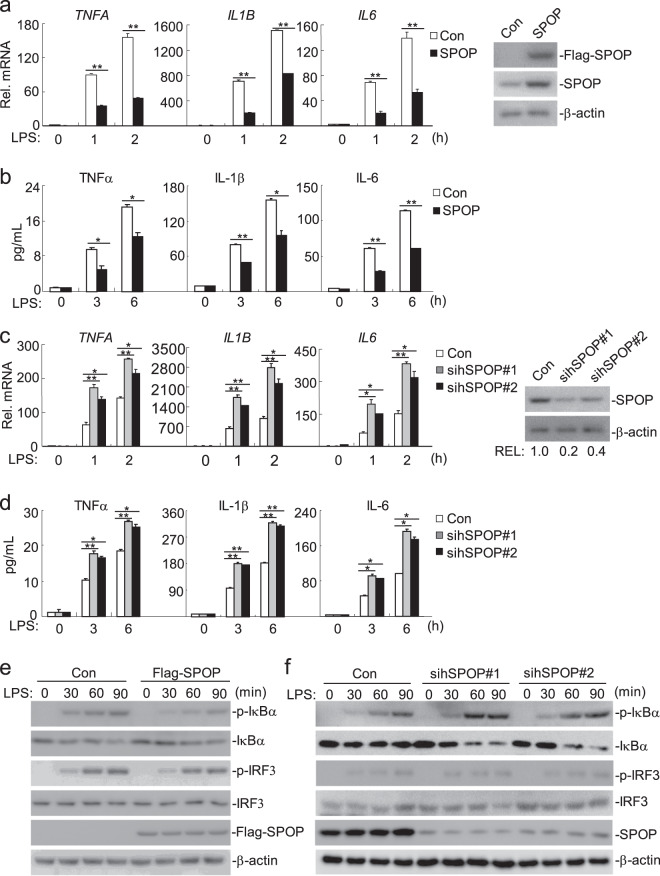
SPOP negatively regulates TLR4-mediated signaling pathways. (**a**) Effects of SPOP on LPS-induced transcription of *TNFA*, *IL1B* and *IL6* genes. THP-1cells were transfected with either an empty control vector or a Flag-SPOP plasmid to establish stable cell lines. Cells (4 × 10^5^) from both stable cell lines were treated with LPS (100 ng/mL) for the indicated times, and then total RNA was prepared for qPCR analysis. Expression of SPOP in the stable cell lines was examined by immunoblot analysis (Right). (**b**) Effects of SPOP on LPS-induced cytokines of TNFα, IL-1β and IL-6. THP-1cells were transfected with either an empty control vector or a Flag-SPOP plasmid to establish stable cell lines. Cells (4 × 10^5^) from both stable cell lines were treated with LPS (100 ng/mL) for the indicated times, and then the medium was collected for ELISA assays. (**c**) Effects of SPOP-RNAi on expression of SPOP and on transcription of *TNFA*, *IL1B* and *IL6* genes. THP-1 cells (4 × 10^5^) were transfected with a control or the indicated SPOP-specific siRNA (2 μg each). Forty hours later, cells were treated with LPS (100 ng/mL) for the indicated times, and then total RNA was prepared for qPCR analysis. The expression of SPOP was examined by immunoblot analysis, and the SPOP bands were quantitated using ImageJ and normalized by levels of the control protein β-actin (Right). (**d**) Effects of SPOP-RNAi on LPS-induced cytokines of TNFα, IL-1β and IL-6. THP-1 cells (4 × 10^5^) were transfected with a control or the indicated SPOP-specific siRNA (2 μg each). Forty hours later, cells were treated with LPS (100 ng/mL) for the indicated times, and then the medium was collected for ELISA assays. (**e**) Overexpression of SPOP inhibits LPS-induced signaling. The control or Flag-SPOP stable expressed cells (4 × 10^5^) were treated with LPS (100 ng/mL) for the indicated times, then subjected to immunoblots with the indicated antibodies. (**f**) Knockdown of SPOP enhances LPS-induced signaling. THP-1 cells (4 × 10^5^) were transfected with a control or the indicated SPOP-specific siRNA (2 μg each). Forty hours later, the cells were treated with LPS (100 ng/mL) for the indicated times and then subjected to immunoblots with the indicated antibodies. Graphs show mean ± SD, *n* = 3. *p < 0.05; **p < 0.01.

Since SPOP interacts with MyD88 following LPS stimulation, we determined whether SPOP is involved in TLR4-mediated signaling. We first established stable Flag-tagged SPOP THP-1 cell lines and performed qPCR and ELISA assays. As shown in Fig. 3a and b, ectopic expression of SPOP inhibited LPS-induced expression of cytokines, including TNFα, IL-1β and IL-6, at both the mRNA and protein levels. Furthermore, overexpression of SPOP significantly inhibited the LPS-induced phosphorylation of IRAK4 and phosphorylation and degradation of IκBα but had no obvious effect on the phosphorylation of IRF3 (Supplementary Fig. S2f and Fig. 3e). Overexpression of SPOP had no marked effect on LPS-induced *IFNB1* transcription (Supplementary Fig. S2a). These data suggest that SPOP specifically inhibits TLR4-mediated NF-κB activation and the expression of downstream inflammatory genes.

### Knockdown of SPOP potentiates TLR4-mediated signaling

Since overexpression of SPOP inhibited TLR4-mediated signaling, we next examined the roles of endogenous SPOP in TLR4 signaling. We designed two small interfering RNAs (siRNAs) for human SPOP (hSPOP siRNA#1 and siRNA#2). As shown in Fig. 3c, siRNA #1 and #2 reduced the endogenous SPOP levels to 20% and 40% those of the control sample, respectively. In the qPCR and ELISA assays, knocking down SPOP markedly potentiated the LPS-induced expression of cytokines, including TNFα, IL-1β and IL-6, at both the mRNA and protein levels in the THP-1 cells, and the degree of promoted expression was correlated with the knockdown efficiencies of the corresponding sihSPOP (Fig. 3c, d). Knockdown of SPOP also increased the LPS- or IL-1β-induced *Tnf*, *Il1b* and *Il6* transcription in the mouse BMDMs (Supplementary Fig. S2c–e). Moreover, knockdown of SPOP markedly increased the LPS-induced phosphorylation of IRAK4 and IκBα and degradation of IκBα, but not IRF3 phosphorylation in the THP-1 cells (Supplementary Fig. S2g and Fig. 3f). Knockdown of SPOP had no marked effect on LPS-induced *IFNB1* transcription (Supplementary Fig. S2b). In addition, knockdown of SPOP markedly increased the LPS-induced transcription of endogenous *Tnf*, *Il1b* and *Il6* and phosphorylation and degradation of IκBα in the RAW264.7 cells (Supplementary Fig. S3a–d). These results suggest that SPOP is a negative regulator of TLR4-mediated NF-κB activation and induction of inflammatory genes.

### Knockout of SPOP potentiates TLR4-mediated signaling

We further generated SPOP-deficient THP-1 cells using CRISPR–Cas9 technology as previously reported.^33^ Two independent SPOP-deficient clones were obtained and confirmed (Supplementary Fig. S4a). When the cells were stimulated with LPS, the transcription of downstream *TNFA*, *IL1B* and *IL6* was significantly increased in both SPOP-deficient cells compared with that of the wild-type cells (Supplementary Fig. S4b).

Reconstitution of SPOP with a SPOP mutant (SPOP-M), containing three nucleotide nonsense mutations in the target sequence of the gRNA, into two SPOP-deficient cells inhibited the promotion of the LPS-induced transcription of downstream genes caused by SPOP knockout (Supplementary Fig. S4c). Furthermore, SPOP-M reconstitution restored the inhibition of the LPS-induced phosphorylation and degradation of IκBα (Supplementary Fig. S4d). Collectively, these data strongly suggest that SPOP is a suppressor of the TLR4-mediated inflammatory response.

### SPOP negatively regulates TLR4 signaling by disrupting MyD88 self-association

**Fig. 4:**
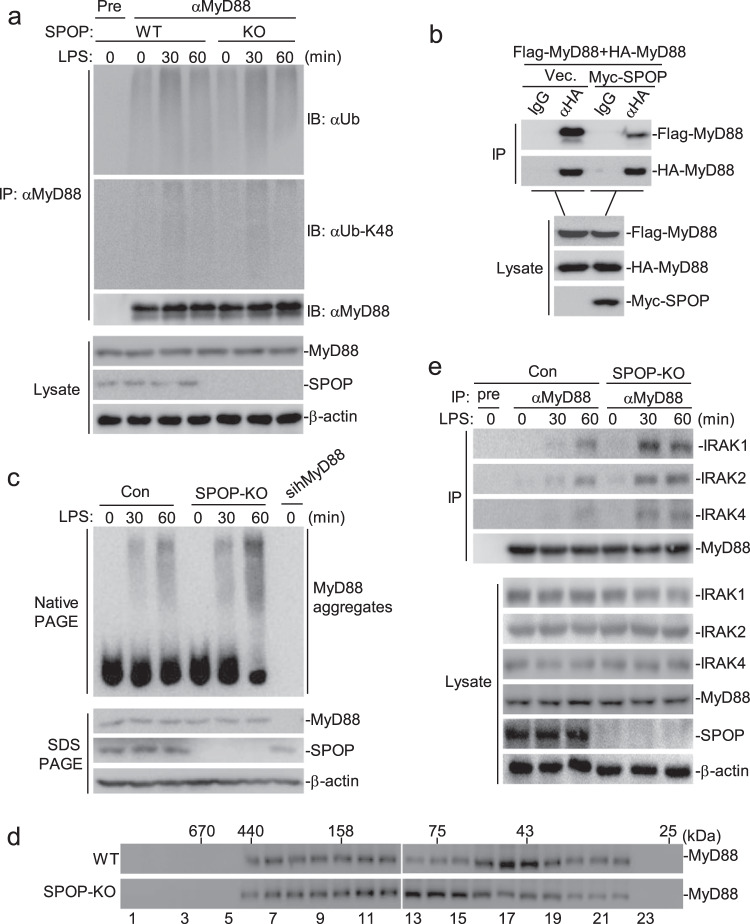
SPOP disturbs MyD88 self-association. (**a**) Effects of SPOP-knockout on ubiquitination of MyD88. The WT or SPOP-deficient THP-1 cells (1 × 10^8^) were treated with LPS (100 ng/mL) for the indicated times. Cells were lysed and the lysates were immunoprecipitated with anti-MyD88. Coimmunoprecipitation and immunoblot analysis were performed with the indicated antibodies. (**b**) SPOP disrupts the self-association of MyD88. HEK293 cells (2 × 10^6^) were transfected with the indicated plasmids (5 μg each) for twenty hours before coimmunoprecipitation and immunoblot analysis with the indicated antibodies. (**c**) SPOP deficiency increases aggregation of MyD88 induced by LPS. The WT or SPOP-deficient THP-1 cells (1 × 10^7^) were treated with LPS (100 ng/mL) for the indicated times, cell lysates were separated by native or SDS PAGE as indicated and analyzed by immunoblots with the indicated antibodies. (**d**) Gel-filtration analysis of complexes containing MyD88 in WT or SPOP-knockout cells. The WT or SPOP-deficient THP-1 cells (1 × 10^8^) were treated with LPS (100 ng/mL) for 1 hour before lysis. Cell lysates were analyzed by size-exclusion chromatography on Superdex 200 column. The individual fractions were analyzed by Western blots with anti-MyD88. (**e**) SPOP deficiency increases LPS-induced recruitment of IRAK1/2/4 to MyD88. The WT or SPOP-deficient THP-1 cells (1 × 10^8^) were treated with LPS (100 ng/mL) for the indicated times. Coimmunoprecipitation and immunoblot analysis were performed with the indicated antibodies.

As an E3 ubiquitin ligase adapter protein, SPOP recruits substrates for ubiquitination and subsequent degradation, and our previous results indicated that SPOP interacts with MyD88 and inhibits the MyD88-mediated signaling pathway. We reasoned that SPOP may mediate MyD88 ubiquitination and degradation. Unexpectedly, overexpression of SPOP failed to catalyze the polyubiquitination of MyD88 or alter the protein levels of MyD88 (Supplementary Fig. S4a, b). Consistent with these findings, SPOP deficiency did not alter MyD88 total polyubiquitination or K48-linked polyubiquitination or expression (Fig. 4a). However, the overexpression of SPOP mediated the polyubiquitination and degradation of PRA, a known SPOP substrate (Supplementary Fig. S4c, d).^28^ Taken together, these results demonstrate that SPOP does not affect MyD88 ubiquitination and degradation.

**Fig. 5:**
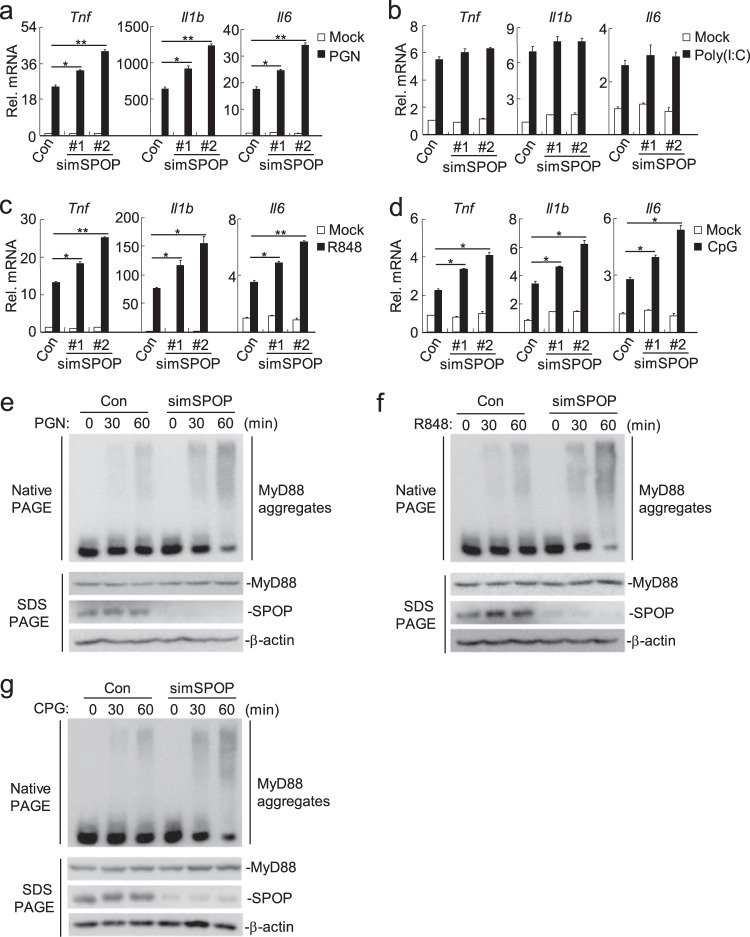
SPOP is specifically involved in the TLR-MyD88-mediated signaling. (**a**) Effects of SPOP on TLR2 signaling. RAW264.7 cells (4 × 10^5^) were transfected with the indicated siRNA (2 μg each) for forty hours. Then the cells were treated with PGN (50 μg/mL) for 2 hours and the total RNA was prepared for qPCR analysis. (**b**) Effects of SPOP on TLR3 signaling. The experiments were similarly performed as in (**a**), except that the stimuli is Poly(I:C) (50 μg/mL). (**c**) Effects of SPOP on TLR7 signaling. The experiments were similarly performed as in (**a**), except that the stimuli is R848 (40 nM). (**d**) Effects of SPOP on TLR9 signaling. The experiments were similarly performed as in (**a**), except that the stimuli is CpG (2 μM). (**e**) Knockdown of SPOP increased aggregation of MyD88 induced by PGN. RAW264.7 cells (2 × 10^6^) were transfected with a control siRNA or SPOP-siRNA#2 (10 μg each) for forty hours, then the cells were treated with PGN (50 μg/mL) for the indicated times. Cell lysates were separated by native- or SDS-PAGE as indicated and analyzed by immunoblots with the indicated antibodies. (**f**) Knockdown of SPOP increased aggregation of MyD88 induced by R848. The experiments were similarly performed as in (**e**), except that the stimuli is R848 (40 nM). (**g**) Knockdown of SPOP increased aggregation of MyD88 induced by CpG. The experiments were similarly performed as in (**e**), except that the stimuli is CpG (2 μM). Graphs show mean ± SD, *n* = 3. *p < 0.05; **p < 0.01.

Previous studies have demonstrated that the self-association of MyD88 is important for its activation after ligand sensing and that it leads to MyD88-induced recruitment of downstream signaling adapters, such as IRAK4, IRAK1, and IRAK2.^7,35^ In transient transfection and coimmunoprecipitation experiments, we found that overexpression of SPOP inhibited the self-association of MyD88 (dimerization and oligomerization) (Fig. 4b). For optimal TLR4-MyD88-dependent signaling, MyD88 adapter-like (Mal) was shown to act as a “bridging molecule” that recruits MyD88 to TLR4.^36^ To investigate whether SPOP affects the recruitment of MyD88 to TLR4, we conducted coimmunoprecipitation assays. The results showed that the overexpression of SPOP had no marked effect on the TLR4-Mal, TLR4-MyD88, or Mal-MyD88 interaction (Supplementary Fig. S5e, f), suggesting that SPOP does not affect the recruitment of MyD88 to TLR4. Furthermore, we sought to determine whether SPOP deficiency affects the endogenous self-association of MyD88. As shown in Fig. 5c, the aggregation of MyD88 induced by LPS was increased in the SPOP-knockout cells compared to that in the wild-type cells. To further confirm these observations, we assessed the regulatory effect of SPOP on the assembly of LPS-induced signaling complexes containing endogenous MyD88 in the presence or absence of SPOP. We found that SPOP deficiency led to a shift in endogenous MyD88 toward fractions of higher molecular weight in the THP-1 cells treated with LPS (Fig. 5d), which confirmed the inhibitory role of SPOP in the assembly of MyD88-mediated signaling complexes.

Consistently, we found that the recruitment of IRAK4, IRAK1, and IRAK2 to MyD88 after LPS stimulation was markedly increased in SPOP-knockout cells (Fig. 5e). These data taken together suggest that SPOP impairs MyD88 self-association and subsequent downstream IRAK recruitment.

### SPOP is specifically involved in TLR-MyD88-mediated signaling

Human SPOP and its mouse ortholog share approximately 94% identity at the amino acid level. We next examined the role of endogenous mouse SPOP (mSPOP) in other TLR signaling pathways in mouse RAW264.7 cells. Knockdown of SPOP promoted PGN (a ligand for TLR2)-, R837 (a ligand for TLR7)- and CpG (a ligand for TLR9)-, but not Poly(I:C) (a synthetic dsRNA ligand for TLR3)-induced transcription of the *Tnf*, *Il1b* and *Il6* genes (Fig. 5a–d). Consistently, knockdown of SPOP increased the aggregation of MyD88 induced by PGN, R837 and CPG (Fig. 5e–g). Given that all TLRs except TLR3 use MyD88 to transmit signals, TLR3 utilizes TRIF. These data suggest that SPOP negatively regulates MyD88-mediated TLR2, TLR7 and TLR9 signaling pathways but not the TRIF-mediated TLR3 signaling pathway.

## Discussion

TLR signaling must be tightly regulated because excessive or uncontrolled TLR signaling causes damage to the host. Despite the pivotal role that MyD88 plays in mediating the pathogen-induced inflammatory response, how MyD88 activity is negatively regulated remains largely unknown. In this report, we identified SPOP, a member of the BTB/POZ family, as a regulator that limits the TLR-mediated inflammatory response through MyD88.

SPOP was previously reported to be located in the nucleus and functions as an E3 ligase adapter to regulate cell development and tumorigenesis by targeting different substrates.^25^ Recently, it was also shown that SPOP is located in the cytoplasm and that cytoplasmic SPOP regulates mitochondrial fission.^34^ In this study, we found that SPOP acted in the cytoplasm in the TLR4-mediated signaling pathway. In the absence of stimulation, SPOP was mainly located in the nucleus but was translocated to the cytoplasm upon LPS stimulation, as revealed by both nuclear and cytoplasmic fractions and confocal microscopy. These observations point to a previously unrevealed cytoplasmic function of SPOP in innate immunity. It is unclear how nuclear SPOP is translocated to the cytoplasm, and further studies are required to address this point.

Using yeast two-hybrid technology, we found that SPOP was associated with MyD88.^32^ Transient transfection and coimmunoprecipitation experiments showed that SPOP interacted with MyD88 constitutively. However, endogenous SPOP only interacted with MyD88 following LPS stimulation. These can be explained by two possibilities. First, overexpression may detect weak interactions. Second, overexpression of MyD88 mimics its activation state, as suggested by its ability to activate NF-κB activation following its overexpression. In fact, in various signal transduction pathways, many protein–protein interactions occur constitutively in the overexpression system, but the endogenous interactions are stimulation-dependent, such as GSK3β-TBK1, WDR5-VISA, and WWP2-TRIF.^37–39^ Moreover, LPS stimulation caused the downregulation of SPOP in the nucleus, suggesting that MyD88-associated SPOP may originate from the nucleus.

To investigate the roles of SPOP in TLR4-mediated signaling, we determined the effects of SPOP on LPS-triggered NF-κB activation and induction of downstream inflammatory genes. We found that overexpression of SPOP inhibited LPS-triggered phosphorylation and degradation of IκBα and induction of inflammatory genes, whereas SPOP depletion had opposite effects. However, SPOP did not affect LPS-triggered phosphorylation of IRF3 and *IFNB1* transcription. These data suggest that SPOP negatively regulates TLR4-mediated NF-κB but not IRF3 activation.

All TLRs except TLR3 utilize MyD88 to ultimately elicit an inflammatory response, whereas TLR3 recruits TRIF to transmit signals. TLR4 uses both MyD88 and TRIF to transmit signals.^15^ Depletion of SPOP enhanced TLR4-mediated inflammatory cytokine expression but not IFN expression. Knockdown of SPOP increased TLR2-, TLR7-, and TLR9-, but not TLR3-mediated production of inflammatory cytokines. IL-1R also uses MyD88 to trigger the inflammatory response. Similarly, knockdown of SPOP increased IL-1β-induced production of inflammatory cytokines. These results suggest that SPOP negatively regulates TLR- or IL-1R-mediated inflammatory cytokine expression through the MyD88-dependent pathway. Specificity in different TLR signaling pathways has been demonstrated with different regulators, such as the adapter protein SRAM, which targets TRIF to inhibit the induction of TLR3- and TLR4-dependent responses.^40^

In our experiments, we found that overexpression of SPOP impaired the self-association of MyD88, whereas knockdown of SPOP increased the aggregation of MyD88 induced by TLR ligands. Domain mapping indicated that the MATH domain of SPOP interacted with the TIR domain of MyD88. The simplest explanation is that the binding of SPOP to MyD88 may cover the TIR domain of MyD88 and prevent the TIR–TIR homotypical interaction of MyD88-self, which leads to the inhibition of MyD88 self-association and activation. Consistently, depletion of SPOP also increased the recruitment of IRAK4, IRAK1, and IRAK2 to MyD88.

Recently, two elegant studies have been published showing SPOP regulating MyD88-NF-κB signaling, one from the Aifantis laboratory and one from the Wang laboratory. The study by Aifantis revealed that SPOP controls the resolution of systemic inflammation by mediating MyD88 ubiquitination and degradation. ^41^ Wang’s study showed that SPOP suppresses the growth of diffuse large B-cell lymphoma by mediating MyD88 nondegradative ubiquitination and blocking myddosome assembly.^42^ Our study suggested that SPOP negatively regulates TLR-triggered inflammation by disrupting MyD88 self-association. Previous studies revealed that one or several SPOP-binding consensus (SBC) motifs are present in the known SPOP substrates.^27^ MyD88 only has one typical SBC motif (14–18 aa); however, neither our group nor that of Aifantis or Wang identified this motif as critical for the association with SPOP. Both the Aifantis and Wang studies suggested that a nontypical motif of MyD88 (135–137 aa) interacted with SPOP, while our findings suggested that the TIR domain of MyD88 (160–296 aa) is important for the association with SPOP. The reason for the different findings remains enigmatic, perhaps because there are other nontypical SBC motifs in the TIR domain of MyD88. Further studies are needed to address this discrepancy. In addition, we identified that the MATH domain in SPOP interacted with MyD88. The observation from the two previously published studies and our data, despite different experimental setups and cell lines, identified the interaction between SPOP and MyD88 underscores the negative regulation of SPOP in MyD88-NF-κB signaling.

Although three of us found that SPOP blocks myddosome assembly and downstream NF-κB activation, the mechanisms for this phenomenon were different. The discrepancy may be due to the following reasons. First, the cell types were different among the groups: Aifantis’s group used K562, 293T, HSPC, and HPC-7 cells; Wang’s group used SU-DHL-2, SU-DHL-4, SU-DHL-6, OCI-Ly3, OCI-Ly10, and Raji cells, and we used HEK293, THP-1, and RAW264.7 cells in our study. Second, the SPOP-mediated K48-linked ubiquitination and degradation of MyD88 were dominant in Aifantis’s cell system, and triggered nondegradative mixed-linkage ubiquitination of MyD88 was dominant in the human lymphoma cell lines used in Wang’s study. It is possible that SPOP functions in different ways in different cell types. Indeed, SPOP targets different substrates and acts as a suppressor in prostate and endometrial cancers and as a promoter in kidney cancer. Therefore, additional studies, especially investigations of the roles of SPOP in other cell types, may resolve this discrepancy. Despite the discrepancies among studies, our findings provide insights into the mechanisms of SPOP-mediated regulation of inflammatory responses.

## Supplementary information

Supplemental Figure Legend

Supplemental Figure S1

Supplemental Figure S2

Supplemental Figure S3

Supplemental Figure S4

Supplemental Figure S5
