# Supplementary material for: SPOP negatively regulates Toll-like receptor-induced inflammation by disrupting MyD88 self-association
Source: Cell Mol Immunol. 2020 Mar 31;18(7):1708–17. doi: 10.1038/s41423-020-0411-1 (PMC8245473; doi:10.1038/s41423-020-0411-1)
Supplement: Supplementary file 1 — Supplemental Figure Legend [file 41423_2020_411_MOESM1_ESM.pdf]

Supplementary Figure S1

(a) Cytoplasmic and nuclear fractions in THP-1 cells. THP-1 cells (3×10^7) were treated with LPS (100 ng/mL) for the indicated times. The cells were fractionated, and the subcellular fractions were equilibrated to equal volumes and analyzed by immunoblots with the indicated antibodies (Upper). The whole cellular levels of SPOP upon LPS stimulation were analyzed by immunoblot with anti-SPOP (Lower).

(b) Cytoplasmic and nuclear fractions in BMDMs. BMDMs (3×10^7) were treated with IL-1β (10 ng/mL) for the indicated times. The cell fractionations and immunoblots were performed as (a).

Supplementary Figure S2

(a) Effects of overexpression of SPOP on LPS-induced transcription of *IFNB1* genes. The control or Flag-SPOP stable expressed THP-1 cells (4×10^5) were treated with LPS (100 ng/mL) for the indicated times, and then cells were harvested and total RNA were extracted for qPCR analysis.

(b) Effects of knockdown of SPOP on LPS-induced transcription of *IFNB1* genes. THP-1 cells (4×10^5) were transfected with a control siRNA or the indicated SPOP-siRNA (2 μg each). Forty hours later, the cells were treated with LPS (100 ng/mL) for the indicated times, and then cells were harvested and total RNA were extracted for qPCR analysis.

(c) Effects of simSPOP on endogenous *Spop* mRNA expression in BMDMs. BMDMs

(4×10^5) were transfected with the indicated siRNA (2 μg each). Forty hours later, total RNA were extracted for qPCR analysis.

(d) Effects of simSPOP on LPS-induced transcription of *Tnf*, *Il1b* and *Il6* genes. BMDMs (4×10^5) were transfected with the indicated siRNA (2 μg each). Forty hours later, the cells were treated with LPS (100 ng/mL) for 2 hours, and then cells were harvested and total RNA were extracted for qPCR analysis.

(e) Effects of simSPOP on IL-1β-induced transcription of *Tnf*, *Il1b* and *Il6* genes. BMDMs (4×10^5) were transfected with the indicated siRNA (2 μg each). Forty hours later, the cells were treated with murine IL-1β (10 ng/mL) for 2 hours, and then cells were harvested and total RNA were extracted for qPCR analysis.

(f) Overexpression of SPOP inhibits LPS-induced signaling. The control or Flag-SPOP stable expressed cells (4×10^5) were treated with LPS (100 ng/mL) for the indicated times, then subjected to immunoblots with the indicated antibodies.

(g) Knockdown of SPOP enhances LPS-induced signaling. THP-1 cells (4×10^5) were transfected with a control or the indicated SPOP-specific siRNA (2 μg each). Forty hours later, the cells were treated with LPS (100 ng/mL) for the indicated times and then subjected to immunoblots with the indicated antibodies.

Graphs show mean ± SD, n = 3. *p < 0.05; **p < 0.01.

Supplementary Figure S3

(a) Effects of simSPOP on endogenous *Spop* mRNA expression in RAW264.7 cells. RAW264.7 cells (4×10^5) were transfected with the indicated siRNA (2 μg each).

Forty hours later, total RNA were extracted for qPCR analysis.

(b) Effects of simSPOP on expression levels of endogenous SPOP protein in RAW264.7 cells. RAW264.7 cells (4×10^5) were transfected with the indicated siRNA (2 μg each). Forty hours later, the expression of SPOP was detected with anti-SPOP.

(c) Effects of simSPOP on LPS-induced transcription of *Tnf*, *Il1b* and *Il6* genes. RAW264.7 cells (4×10^5) were transfected with the indicated siRNA (2 μg each). Forty hours later, the cells were treated with LPS (100 ng/mL) for 2 hours, and then cells were harvested and total RNA were extracted for qPCR analysis.

(d) Knockdown of SPOP enhances LPS-induced signaling. RAW264.7 cells (4×10^5) were transfected with the indicated siRNA (2 μg each). Forty hours later, the cells were treated with LPS (100 ng/mL) for the indicated times, and then subjected to immunoblots with the indicated antibodies.

Graphs show mean ± SD, n = 3. *p < 0.05; **p < 0.01.

Supplementary Figure S4

(a) Identification of SPOP-deficient cells. SPOP-deficient and control THP-1 cells (2×10^6) were harvested and lyzed. The immunoblot analysis was performed with the indicated antibodies.

(b) Effects of SPOP deficiency on LPS-induced transcription of *TNFA*, *IL1B* and *IL6* genes. The indicated cells (4×10^5) were treated with LPS (100 ng/mL) for 2 hours and then the total RNA was prepared for qPCR analysis.

(c) Effects of LPS-induced transcription of downstream genes in SPOP-deficient cells reconstituted with SPOP-M. SPOP-deficient THP-1 cells were reconstituted with an empty vector or SPOP-M by lentiviral-mediated gene transfer. Expression of SPOP in cells was examined by immunoblot analysis (Left). The indicated cells (4×10^5) were treated with LPS (100 ng/mL) for 2 hours, and then the total RNA was prepared for qPCR analysis.

(d) Effects of LPS-induced signaling in SPOP-deficient cells (#1) reconstituted with SPOP-M. The indicated cells (4×10^5) were treated with LPS (100 ng/mL) for the indicated times and then subjected to immunoblots with the indicated antibodies. Graphs show mean $\pm$ SD, n = 3. $^{**}p < 0.01$.

Supplementary Figure S5

(a) Effects of SPOP on ubiquitination of MyD88. HEK293 cells (2×10^6) were transfected with the indicated plasmids (5 μg each) for twenty hours before immunoprecipitation and immunoblotting analysis with the indicated antibodies.

(b) SPOP does not affect the expression of MyD88. HEK293 (4×10^5) cells were transfected with the indicated plasmids for twenty hours and then immunoblots were performed with the indicated antibodies.

(c) Effects of SPOP on the ubiquitination of PRA. HEK293 cells (2×10^6) were transfected with the indicated plasmids (5 μg each) for twenty hours before immunoprecipitation and immunoblotting analysis with the indicated antibodies.

(d) SPOP reduces the expression of PRA. The experiments were performed as in (b).

(e) Effects of SPOP on TLR4-Mal-MyD88 interaction. HEK293 cells (2×10^6) were transfected with the indicated plasmids (5 μg each) for twenty hours before coimmunoprecipitation and immunoblotting analysis with the indicated antibodies.

(f) Effects of SPOP on Mal-MyD88 interaction. The experiments were performed as in (e).
