# Supplementary figures and images for: SPOP negatively regulates Toll-like receptor-induced inflammation by disrupting MyD88 self-association
Source: Cell Mol Immunol. 2020 Mar 31;18(7):1708–17. doi: 10.1038/s41423-020-0411-1 (PMC8245473; doi:10.1038/s41423-020-0411-1)

# Supplementary Figure S1

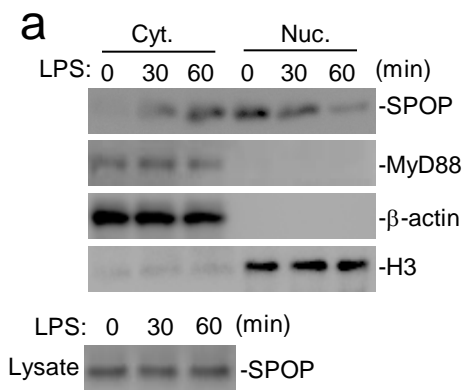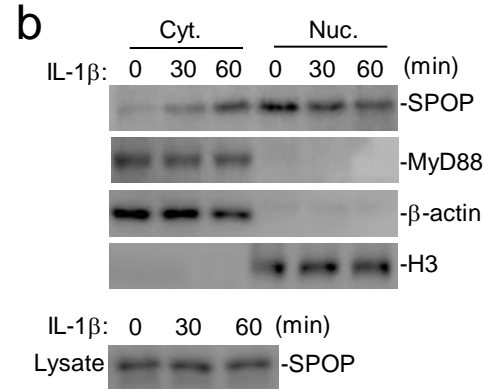

Supplement: Supplementary file 2 — Supplemental Figure S1 [file 41423_2020_411_MOESM2_ESM.pdf]

Supplementary Figure S2

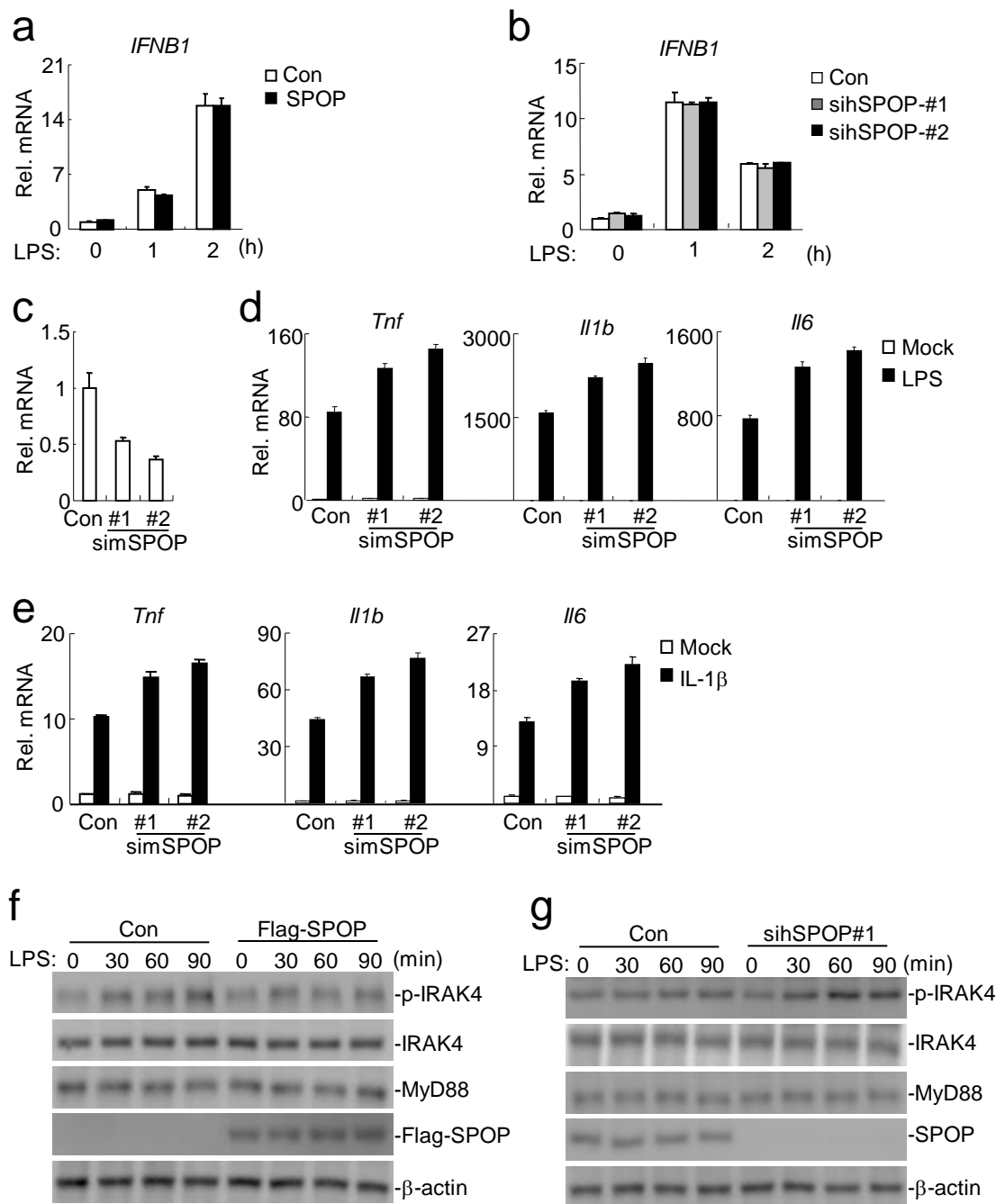

Supplement: Supplementary file 3 — Supplemental Figure S2 [file 41423_2020_411_MOESM3_ESM.pdf]

Supplementary Figure S3

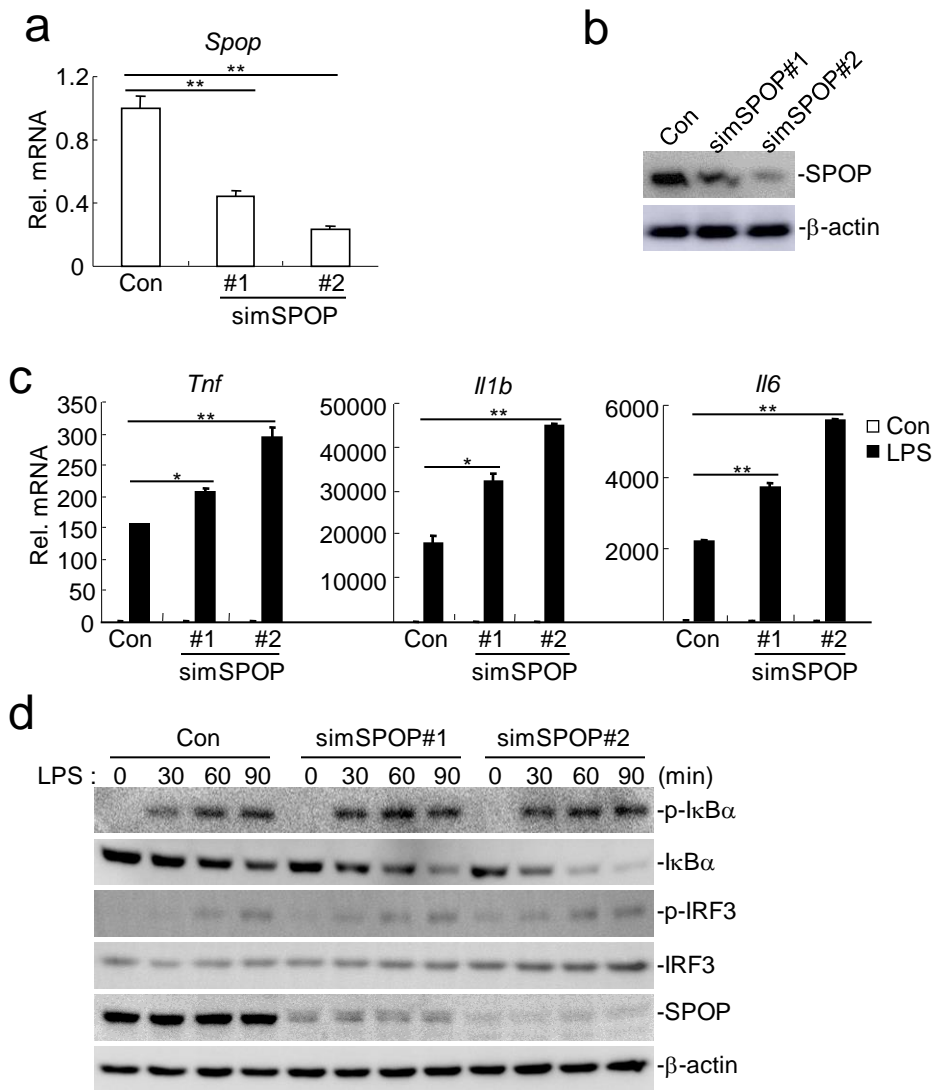

Supplement: Supplementary file 4 — Supplemental Figure S3 [file 41423_2020_411_MOESM4_ESM.pdf]

Supplementary Figure S4

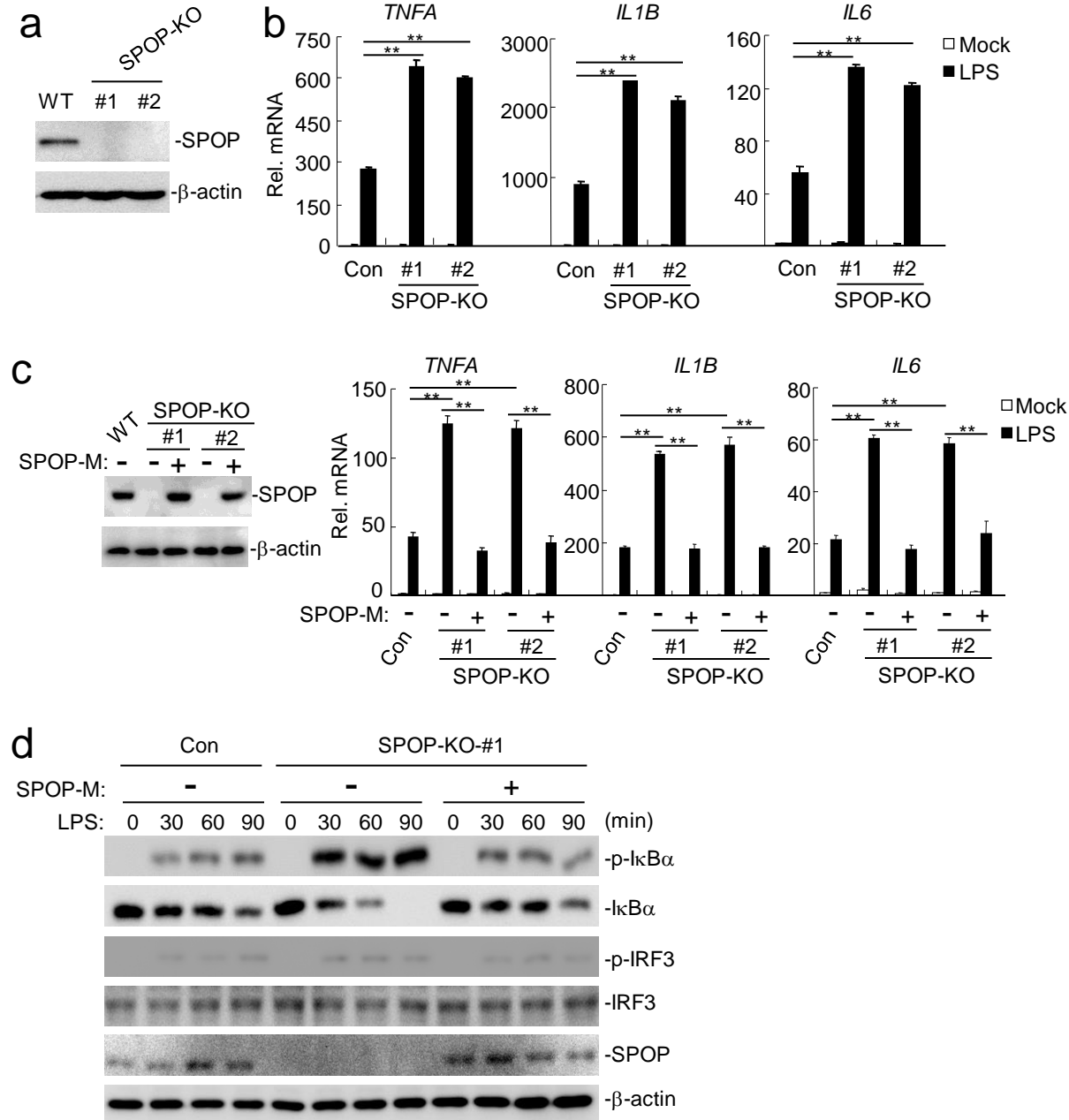

Supplement: Supplementary file 5 — Supplemental Figure S4 [file 41423_2020_411_MOESM5_ESM.pdf]

Supplementary Figure S5

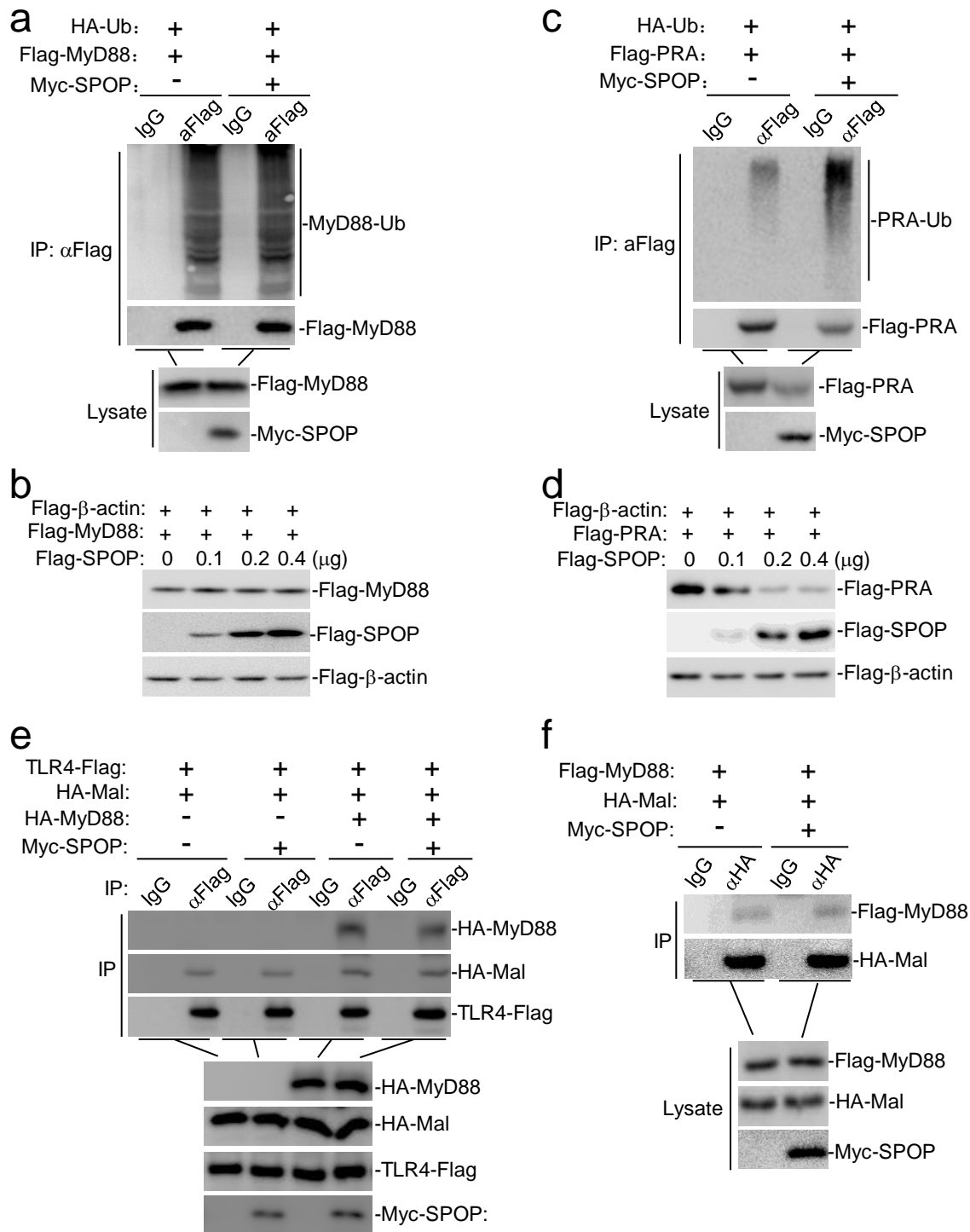

Supplement: Supplementary file 6 — Supplemental Figure S5 [file 41423_2020_411_MOESM6_ESM.pdf]
